# Genoprotective, antioxidant, antifungal and anti-inflammatory evaluation of hydroalcoholic extract of wild-growing *Juniperus communis* L. (*Cupressaceae*) native to Romanian southern sub-Carpathian hills

**DOI:** 10.1186/s12906-017-2066-8

**Published:** 2018-01-04

**Authors:** Irina Fierascu, Camelia Ungureanu, Sorin Marius Avramescu, Carmen Cimpeanu, Mihaela Ioana Georgescu, Radu Claudiu Fierascu, Alina Ortan, Anca Nicoleta Sutan, Valentina Anuta, Anca Zanfirescu, Cristina Elena Dinu-Pirvu, Bruno Stefan Velescu

**Affiliations:** 1 The National Institute for Research & Development in Chemistry and Petrochemistry, ICECHIM, 202 Spl. Independentei, 060021 Bucharest, Romania; 2 University of Agronomic Science and Veterinary Medicine, 59 Marasti Blvd, 011464 Bucharest, Romania; 3 Faculty of Applied Chemistry and Material Science, University Politehnica of Bucharest, 1 Polizu Str., 011061 Bucharest, Romania; 4 Research Center for Environmental Protection and Waste Management, University of Bucharest, 36-46 M. Kogalniceanu Blvd., 050107 Bucharest, Romania; 5 Department of Natural Sciences, University of Pitesti, 1 Targu din Vale, 110040 Pitesti, Arges Romania; 6 Faculty of Pharmacy, Carol Davila University of Medicine and Pharmacy, 6 Traian Vuia Str., 020956 Bucharest, Romania

**Keywords:** Natural compounds, Hydroalcoholic extract, Genoprotective

## Abstract

**Background:**

*Juniperus communis* L. represents a multi-purpose crop used in the pharmaceutical, food, and cosmetic industry. Several studies present the possible medicinal properties of different Juniperus taxa native to specific geographical area. The present study aims to evaluate the genoprotective, antioxidant, antifungal and anti-inflammatory potential of hydroalcoholic extract of wild-growing *Juniperus communis* L. (*Cupressaceae*) native to Romanian southern sub-Carpathian hills.

**Methods:**

The prepared hydroethanolic extract of *Juniperus communis* L. was characterized by GC-MS, HPLC, UV-Vis spectrometry and phytochemical assays. The antioxidant potential was evaluated using the DPPH assay, the antifungal effect was studied on *Aspergillus niger* ATCC 15475 and *Penicillium hirsutum* ATCC 52323, while the genoprotective effect was evaluated using the *Allium cepa* assay. The anti-inflammatory effect was evaluated in two inflammation experimental models (dextran and kaolin) by plethysmometry. Male Wistar rats were treated by gavage with distilled water (negative control), the microemulsion (positive control), diclofenac sodium aqueous solution (reference) and microemulsions containing juniper extract (experimental group). The initial paw volume and the paw volumes at 1, 2, 3, 4, 5 and 24 h were measured.

**Results:**

Total terpenoids, phenolics and flavonoids were estimated to be 13.44 ± 0.14 mg linalool equivalent, 19.23 ± 1.32 mg gallic acid equivalent, and 5109.6 ± 21.47 mg rutin equivalent per 100 g of extract, respectively. GC-MS characterization of the juniper extract identified 57 volatile compounds in the sample, while the HPLC analysis revealed the presence of the selected compounds (α-pinene, chlorogenic acid, rutin, apigenin, quercitin). The antioxidant potential of the crude extract was found to be 81.63 ± 0.38% (measured by the DPPH method). The results of the antifungal activity assay (for *Aspergillus niger* and *Penicillium hirsutum*) were 21.6 mm, respectively 17.2 mm as inhibition zone. Test results demonstrated the genoprotective potential of *J. communis* undiluted extract, inhibiting the mitodepressive effect of ethanol. The anti-inflammatory action of the juniper extract, administered as microemulsion in acute-dextran model was increased when compared to kaolin subacute inflammation induced model.

**Conclusion:**

The hydroalcoholic extract obtained from wild-growing *Juniperus communis* native to Romanian southern sub-Carpathian hills has genoprotective, antioxidant, antifungal and anti-inflammatory properties.

## Background

Natural products (extracts or essential oils) obtained from various plants are complex mixtures, containing hundreds of organic compounds that are usually used as food, beverages, flavouring and aroma agents [1]. These natural products are currently promoted as anticancer, anti-diabetic, antibacterial, antiviral and antioxidant agents, and in various other applications (such as the phytosynthesis of nanoparticles) [2–7]^.^ Most of these therapeutic activities mentioned could be attributed to polyphenolic compounds found in natural products [8].

*Juniperus communis* L. is an evergreen tree growing in many regions in Eurasia, North Africa and North America. From the *Juniperus* L. genus, consisting of 67 species and 34 varieties, the most common juniper species in Central and Southeast Europe is *Juniperus communis* L., which can be identified based on macroscopic and microscopic differences compared to other species of juniper [9, 10]. Its usable parts (berries – *Juniperi fructus* and needles – *Juniperi foliage*) contain an essential oil with a characteristic and recognizable flavour. The main value of juniper as a crop resides in the application of its essential oil and extracts as diuretic, in gastrointestinal diseases, renal, genital, pulmonary and rheumatic disorders, in pharmaceutical and food industries, perfumery or in cosmetics [11].

Recent papers studied the use of juniper extracts natural products (essential oil or extracts) mainly as antioxidants [12] and antimicrobial agents [13, 14]. Their hypoglycaemic and hypolipidemic effects and cytotoxic activity were also investigated [15]. The *anti-inflammatory* potential of juniper was empirically established and transmitted in the folk medicine of different countries, throughout Europe [11, 16, 17]. Scientific evidences of the anti-inflammatory effect of several *Juniperus* taxa are provided by many in vitro and in vivo studies published in the last decades. Mascolo et al. [16] evaluated 75 (most frequently used in Italian folk medicine) hydro-alcoholic plant extracts for the in vivo anti-inflammatory activity using carrageenan foot oedema model. Among them, *Juniperus communis* L. qualified in the first four species, considering their activity. Tunon et al. [17] evaluated the anti-inflammatory potential of 59 water extracts (obtained from Swedish medicinal plants) using in vitro assays. Once again, the juniper extract was found to be active in both assays used (prostaglandin biosynthesis and PAF-induced exocytosis). Akkol et al. [18] evaluated five Turkish *Juniperus* taxa methanolic and aqueous extracts for anti-inflammatory activity in carrageenan-induced and PGE_2_-induced hind paw oedema model, offering scientific support for their traditional use. Kalinkevich et al. [19] included their in vitro study regarding the anti-inflammatory activities of 133 plants, vegetables, fruits and mushrooms native to Russia, the ethanolic extract obtained from *Juniperus communis* L. Their results situated the juniper extract as having an average anti-inflammatory potential. Other *Juniperus* taxa, such as *Juniperus sibirica* Burgsdorf. [20], *Juniperus foetidissima* Willd. 1806 [21] or *Juniperus macrocarpa* Sibth. et Sm. [22] (native to Serbia) were evaluated by in vitro assays, with very good results. The literature data presented suggests that further investigations are necessary to verify and establish the anti-inflammatory effect, especially considering the variations between vegetal materials. In Romania, juniper fruits are traditionally used as infusion or tincture, both internally (as diuretic and antiseptic) and externally (for various dermatitis conditions) [11].

Considering the various factors affecting the final composition of natural extracts [23–25], it is not only possible but even probable that different researchers will obtain different results for the same plant species.

The objectives of the study were the preparation, chemical characterisation and the assessment of antioxidant, antifungal, genoprotective and anti-inflammatory properties of hydroalcoholic extract of wild-growing *Juniperus communis* L. (*Cupressaceae*).

## Methods

### Plant material and extraction technique

Wild-growing *Juniperus communis* L. was obtained from Dobresti area, Pitesti hills (Romanian southern sub-Carpathian hills, 44°57′48″N, 25°6′58″E, 450 m above sea level) in August 2014. Multiple plants were identified at the harvesting site; from those, two representative voucher specimens were deposited in BUAG Herbarium, Bucharest for future reference (voucher nos. 40,003 and 40,004). Plant materials were formally identified by Mihaela Ioana Georgescu, PhD, Associate Professor at the Department of Horticulture, University of Agronomic Science and Veterinary Medicine.

Fruits were carefully collected over a period of 3 weeks from multiple individual vegetal sources, selecting the ripe ones, as fruits in all stages of a multi-annual ripening cycle (which covers a period of approx. 18 months) are usually found on the same plant [26], aiming to obtain a representative harvest for the specific area.

The *Juniperus communis* L. extract used for the study was obtained from 20 g of ground shade-dried fruits using 200 mL of solvent (water-ethanol 1:1 mixture), as previously described by our group [6, 25]. The experiments were carried out using analytic grade ethanol (Merck KGaA, Germany), and bidistilled water obtained using a GFL 2102 water still.

### Analytical characterisation methods

In order to evaluate its chemical composition, the extract was characterized using UV-Vis spectrometry, gas chromatography–mass spectrometry and high-performance liquid chromatography.

### Instruments conditions

An UV-Vis spectrometer Unicam Helios α Thermo Orion was used to acquire scans from 200 to 900 nm (resolution 1 nm, 1 nm slit width, automatic scan rate), in order to obtain extraction factor and to perform phytochemical analyses. Results were processed with specific data analysis software (Origin Pro 8.0). The extraction factor was obtained from the equation:

1 $$ \mathrm{EF}={\mathrm{A}}_{\uplambda \mathrm{max}}\times \mathrm{DF} $$

*where* EF – extraction factor, A_λmax_- absorption values, DF- dilution factor [25, 27]. The experiments were carried out in triplicate.

A Varian 3800 gas chromatograph coupled to a Varian 2000 mass spectrometer (GC–MS) with FID detector was used to analyse the natural products, using for analytes separation an FactorFour WCOT fused silica column (stationary phase: VF-624 ms; column length: 30 m; inside diameter: 0.25 mm; film thickness: 1.40 μm) supplied by Varian Inc.

The following conditions were used: column temperature from 50 °C (held for 1 min) to 280 °C (held for 10 min) at a rate of 6 °C min^−1^; injector temperature, 200 °C; injection mode, split mode (20); helium carrier gas flow rate 1.0 mL min^−1^; MS transfer temperature, 280 °C; ion source temperature, 250 °C; ionization mode, electron impact; ionization energy, 70 eV; mass scan range, *m*/*z* 50–650. The results were analysed and interpreted using specific software and the NIST98 Mass Spectral Database. Before injection, the extract was first evaporated using a rotary evaporator and then diluted using a non-polar solvent (hexane, 1 g/10 mL of solvent).

The HPLC analyses were carried out using a Varian system consisting of a solvent delivery pump (Prostar 410), a DAD detector (Prostar 335) and an autosampler (Prostar 410) with a partial loop-fill volume. Data collection and analyses were performed using Varian Workstation 6.3 software.

Working procedure involves a gradient elution performed on a *Zorbax eclipse plus C18* column (150 × 4.6 mm i.d., 5 μm particle size) (Agilent). The mobile phase consisted of two different solutions, solution A (1% acetic acid in water) and solution B (1% acetic acid in acetonitrile).

All solutions were degassed and filtered through a 0.45 μm pore size filter (LABTECH VP30). Separations were performed using a gradient elution procedure as follows: from 0 to 90 min, solution B followed a linear change from 5% to 100% and from 90 to 95 min, B was isocratic at 100. The flow rate was 1 mL min^−1^ and the injection volume was 10 μL. UV detection was performed at 276 nm.

Using these chromatographic conditions, it was possible to confirm the retention time of analytes. Five-points calibration curves were constructed for each of the compounds analysed (R^2^ > 0.999) using commercial available standard materials (Merck KGaA, Germany). The selected compounds belong to several types: terpenoid (*α-pinene*), phenolic acid (*chlorogenic acid*) and flavonoids (*rutin, apigenin, quercitin*).

For the study of the microemulsion formulations, conductibility studies were performed using a Corning 441 conductivity meter (Corning, NY, USA). The refractive index was determined at 25 °C using a digital Abbe refractometer. The mean diameter of the droplets and the Zeta potential of the microemulsion were measured using a Mastersizer 2000 (Malvern, UK) particle size analyser.

### Phytochemical assays

For the phytochemical evaluation of the extract were used specific procedures for the determination of total phenolics content [28], total flavonoids [29] and total terpenoids [30], as described in detail in previous studies [6, 25]. The calibration curves were constructed using analytic standards (gallic acid, rutin and, respectively, linalool, Sigma-Aldrich, Germany) The experiments were carried out in triplicate and the results are presented as standard equivalents.

### Antioxidant assay

The antioxidant activity was determined using the DPPH (2,2-diphenyl-1-picrylhydrazyl assay, as previously described [25]. The antioxidant activity was calculated from the decrease of absorbance upon sample addition to the DPPH solution, using the formula:

2 $$ \mathrm{AA}\left(\%\right)=\left[\left({\mathrm{A}}_{\mathrm{control}}-{\mathrm{A}}_{\mathrm{sample}}\right)/{\mathrm{A}}_{\mathrm{control}}\right]\times 100\Big) $$

*where:* AA (%) is the antioxidant activity (in percent), A_control_ is the absorbance of the DPPH solution and A_sample_ is the absorbance of the extract mixed with DPPH solution.

The half maximal effective concentration (EC_50_) was calculated using specialized data analysis software (Origin Pro 8.0) [31] and evaluated by comparison with one known antioxidant (*ascorbic acid*, Sigma-Aldrich). All the experiments were carried out in triplicate.

### Determination of antifungal effect

The antifungal activity was evaluated using the disc diffusion method [32–34]. The antifungal activity was tested against *Aspergillus niger* (ATCC 15475) and *Penicillium hirsutum* (ATCC 52323) fungal strains, cultivated onto potato-dextrose agar (PDA) sterile plates (Sigma-Aldrich). One mL of test organism was spread on the plates. Wells were made using a sterile Durham tube of 6 mm diameter, and were inoculated with 50 μL of hydroalcoholic extract. As negative control was used the solvent used for extraction (ethanol: H_2_O = 1:1), while as positive control was used *miconazole nitrate* solution (30 μg/mL, Sigma-Aldrich). The plates were incubated at 37 °C for 84 h.

The antifungal activity was determined from the sizes of inhibition zone (IZ, mm), considering values under mm in diameter as not active. The percent inhibition percent was calculated using the formula:

3 $$ \mathrm{I}\left(\%\right)=\left[\left(\mathrm{IZ}-\mathrm{NC}\right)/\mathrm{IZ}\right]\times 100 $$

*where* I – inhibition percent, IZ - inhibition zone diameter using the extract and NC – inhibition zone for the negative control.

The data was analysed for statistical significance using analysis of variance (one-way ANOVA) and Tukey test was used to determine significant differences among means. Significant differences were set at *P ≤ 0.05*. The results presented represent the *Mean ± standard error of mean (SEM)* of independent replicates.

### Evaluation of cytogenetic effects

The juniper extract mitostimulatory and antimutagenic potential was evaluated by monitoring the changes in mitotic index (MI) and phase indexes (prophase, metaphase, anaphase, telophase), and chromosomal aberrations frequency in root tips cells of *Allium cepa* L. [7].

Onions (local variety) were purchased from local market. Eighteen healthy onion bulbs were used in the experiments by removing the outer scales and scrapping the bottoms to expose root primordia. In order to induce rhizogenesis and root growth, the bulbs were placed on 30 mL jars filled with distilled water. After 48 h, the roots, freshly emerged, were treated with hydro-alcoholic extracts 5%, 25%. 50% and 100% for 48 h. Tap water served as negative control and solvent (water: ethanol = 1:1) as positive control.

Cytological analyses were performed on squash slides prepared following the protocol of Tedesco and Laughinghouse [35]. About 3000 cells from 9 root tips were scored for each treatment. The cells at different stages of mitosis were noticed.

Mitotic index (MI) was computed by determining the mitotic cell frequency (prophase, metaphase, anaphase and telophase) by the total number of cells observed and multiplying the result by 100 [35]. The number of cells at various mitosis stages (prophase, metaphase, anaphase and telophase) was calculated as percentage to number of dividing cells. The abnormality percentage was recorded as the percentage of abnormally divided cells in the appropriate mitotic stage. Photomicrographs of cells showing chromosomal aberrations, as well as showing normal mitosis, were taken using Olympus CX-31 microscope at 400× magnification.

Results are presented as the *Mean ± standard error* of several independent experiments. The data was analysed for statistical significance using analysis of variance (one-way ANOVA) and Duncan test was used to determine significant differences among means. Significant differences were set at *P ≤ 0.05*.

### Microemulsion preparation

Due to the general low bioavailability of the polyphenolic compounds, the extract was formulated as a *microemulsion* and tested for *anti-inflammatory action* [36, 37]*.*

Equilibrium solubility experiments were carried out in order to select the appropriate oil, surfactant, and cosurfactant constituents of the microemulsion. An excess amount of extract was added to 5 mL of oil, surfactant or co-surfactant, and the resulting mixture was stirred for 30 s at 2500 rpm on an IKA Genius 3 vortex mixer (IKA Werke GmbH & Co. KG, Staufen, Germany). The mixture was then shaken (250 rpm) at room temperature for 24 h on a IKA HS 260 orbital shaker (IKA Werke GmbH & Co. KG, Staufen, Germany), followed by centrifugation for 10 min at 12,000 rpm on a Hettich Mikro 220R centrifuge (Andreas Hettich GmbH & Co. KG, Tuttlingen, Germany). The supernatant was filtered through a 0.45 μm Teflon® filter, and UV spectra were recorded after suitable dilution. Based on preliminary solubility experiments, oleic acid, Tween 80 and propylene glycol were selected as the oil phase, surfactant, and as cosurfactant, respectively.

The behaviour of the multi-component microemulsion system was studied by constructing pseudo-ternary phase diagrams. The ratio of surfactant to co-surfactant was fixed at 1:1 based on their weights. Oleic acid was mixed with the surfactant: co-surfactant mixture at ratios of 1:9, 2:8, 3:7, 4:6, 5:5. 6:4, 7:3, 8:2, 9:1, and distilled water was added to the mixture in increments of 100 μL by micropipette under vigorous shaking. In order to reach equilibrium prior to further evaluation, the resulting samples were maintained at 25 °C for 24 h. The mixtures were then visually assessed and samples that remained homogeneous and visually transparent were selected as microemulsions. The same procedure was applied when preparing microemulsions containing *Juniperus communis* L. extract, with the extract being dispersed into the surfactant: cosurfactant mixture.

### Determination of the anti-inflammatory effect

*Male Wistar rats* weighing 212 ± 45 g from the University of Medicine and Pharmacy, Bucharest animal facility (rodent farm) were used for the in vivo studies. The specimens used for experiments were housed in plastic cages (1354G EUROSTANDARD type IV), fed with granulated food, free access to water. Temperature was kept between 20 and 22 °C, while the relative humidity was maintained at 35-45%. Inflammation was evaluated in *two inflammation experimental* models by plethysmometry (Ugo Basile 7140 Plethysmometer), as previously reported [38].

Inflammation was induced by intraplantar administration of 0.2 mL inflammatory agent (0.6% solution of dextran and 10% aqueous suspension of kaolin, respectively) into the rat’s inferior right paw. The anti-inflammatory effect was compared with a negative control group (untreated rats), a positive control group (rats treated with the microemulsions vehicle) and a reference substance (diclofenac) group. The two models were selected as dextran and kaolin induced inflammation have different pathways (dextran is histamine and serotonin mediated, while kaolin is proinflammatory cytokine mediated).

Male Wistar rats were put into 8 groups (*n* = 8) and treated with 10 mL/kg body weight (b.w.) distilled water (negative control), 10 mL/kg b.w. of the microemulsion (positive control), 100 mg/kg b.w. diclofenac sodium, or 10 mL/kg b.w. of microemulsions containing juniper extract by gavage. After drug administration, the initial paw volume and the paws volumes at 1, 2, 3, 4, 5, and 24 h after the administration of the inflammatory agent were taken after being anesthetized by intraperitoneal injection of 130 mg/kg b.w. urethane. The research involving animal experiments was conducted in accordance with the European Community guidelines (2010/63/EU) and had the approval of the local ethics committee (“Carol Davila” Medicine and Pharmacy University, Faculty of Pharmacy, Bioethics Commission – approval no. 2086/2017).

*The statistical analyses* were performed using GraphPad Prism 7 software. The evolution of paw oedema was calculated using the formula:

4 $$ \%=\left[\left({\mathrm{V}}_{\mathrm{xh}}-{\mathrm{V}}_0\right)/{\mathrm{V}}_0\right]\times 100 $$

*where* V_0_ is the initial paw volume and V_*xh*_ is the paw volume at each time measurement. The anti-inflammatory effect was calculated as the difference between the evolutions of paw edema of the treated groups and the negative control group or the reference group. Results are expressed as mean ± standard deviation. The experiments were carried out in accordance with ARRIVE guidelines [39]. The applied parametrical tests (t test, one-way ANOVA) have a 90% confidence interval and statistical differences were considered for *p* value <0.05.

In order to apply the parametrical tests, the normal distribution of the results was verified with Kolmogorov-Smirnov normality test. If the results did not pass the test, outliers were identified and excluded based on Dixon criteria.

## Results

The major problem that stop the inclusion of medicinal plants in the International Pharmacopoeias, is the lack of research that validate their use through the chemical and pharmacological characterization of the final extracts [40]. In this sense, Aarland et al. [41] reported that the most important step in order to develop phytopharmaceutical products from medicinal plants is the standardization of the extracts.

### Evaluation of the extract’s composition

**Fig. 1:**
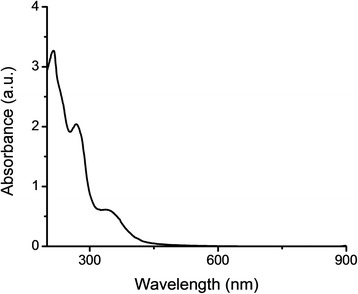
UV-VIS spectrum of diluted extract (dilution factor = 100)

To inspect the absorption characteristics of the sample, the UV-Vis spectra of the diluted extract were recorded (Fig. 1 represents the spectrum recorded for a dilution factor of 100, used to determine the extraction efficiency in the region 290-420 nm). The analyses were focused in the regions were phenolic acids and its derivatives (flavones, flavonols, phenylpropenes, quinones) present specific absorption peaks (220-280 nm and, respectively, 290-420 nm) [27]. Table 1 presents the specific absorption values for the plant extract, as well the extraction efficiency (EF factor), calculated according to formula (1).

**Table 1:** Specific absorption values for the extract and EF calculated values

Dilution factor (DF)	A_220-280nm_	EF_220-280 nm_	A_290-420 nm_	EF_290-420 nm_
DF10	–	–	–	–
	–	–	–	–
DF100	–	–	A_416_ = 0.1128	11.28
	–	–	A_350_ = 0.5994	59.94
DF1000	A_222_ = 0.5116	511.6	–	–
	A_267_ = 1.2215	1221.5	–	–

Phytochemical evaluations of the crude extract were performed by spectrophotometric methods. The results and calibration curves parameters are presented in Table 2 [6].

**Table 2:** Results and calibration curves parameters of the phytochemical assays

No.	Assay	Standard	Curve parameters (Equation/R^2^)	Results (mg equivalents/100 g extract)
1	Total phenolics content	Gallic acid	y = 0.01122x + 0.00804, R^2^ = 0.9979	19.23 ± 1.32
2	Total flavonoids	Rutin	y = 0.0067x-0.0401, R^2^ = 0.996	5109 ± 0.02
3	Total terpenoids	Linalool	y = 0.0016x + 00168, R^2^ = 0.993	13.44 ± 0.14

**Fig. 2:**
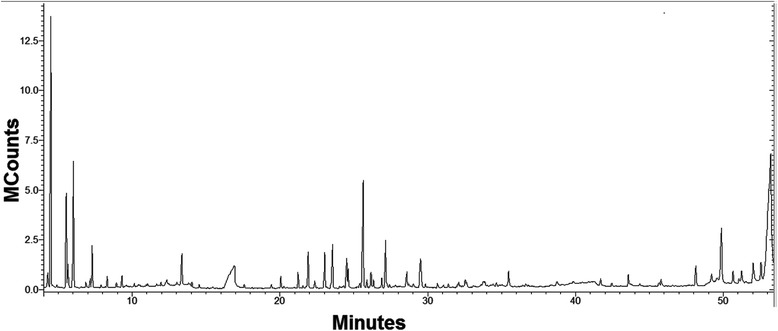
GC-MS chromatogram of the juniper extract

GC-MS characterization of the juniper extract identified 57 volatile compounds in the sample. The GC-MS chromatogram is shown in Fig. 2, while the major (peak area > 0.3%) identified components (based on the highest probability) are summarized in Table 3 and trace elements (peak area ≤ 0.3%) are presented in Table 4.

**Table 3:** Major and minor compounds identified by GC-MS in the juniper extract (over 0.3% peak area)

No.	Compound	Retention time (min.)	Peak area (%)
1	3-Carene	4.314	0.76
2	α-Pinene	4.508	12.84
3	β-Pinene	5.555	4.57
4	β-Myrcene	6.057	6.04
5	D-Limonene	7.299	1.64
6	Terpinolene	8.314	0.34
7	4-Carene	9.312	0.39
8	Terpinen-4-ol	13.370	1.45
9	α-Cubebene	20.063	0.46
10	Copaene	21.234	0.62
11	(−)-β-Elemene	21.912	1.53
12	Caryophyllene	23.035	1.82
13	γ-Elemene	23.552	2.12
14	α-Humulene	24.517	1.23
15	trans-β-Farnesene	24.632	0.87
16	Bicyclosesquiphellandrene	25.647	5.94
17	β-Selinene	25.890	0.31
18	α-Selinene	26.167	0.64
19	α-Muurolene	26.346	0.36
20	γ-Cadinene	26.892	0.43
21	β-Cadinene	27.150	2.12
22	Eremophilene	28.582	0.75
23	Germacrene D-4-ol	29.510	2.15
24	Juniper camphor	32.540	0.43
25	γ-Selinene	33.763	0.34
26	α,2,6,6-Tetramethyl-1-cyclohexene-1-methanol	35.481	0.87
27	Biformene	41.702	0.36
28	Verticiol	43.589	0.47
29	Manool	45.806	0.43
30	Sclarene	48.134	1.14
31	Isopimara-7,15-dien-3-one	49.885	3.93
32	Pimaric acid	53.226	25.70
TOTAL			83.05

**Table 4:** Trace compounds identified by GC-MS in the juniper extract (area ≤ 0.3%)

No.	Compound	Retention time (min.)	Peak area (%)
1	Camphene	4.900	0.12
2	1,5,5-Trimethyl-6-methylenecyclohexene	6.879	0.11
3	m-Cymene	7.169	0.30
4	Myrtenol	7.894	0.12
5	5-Caranol	8.941	0.16
6	Cis-para-2-menthen-1-ol	10.155	0.09
7	6-Camphenol	11.050	0.12
8	Trans-para-2-menthen-1-ol	10.952	0.06
9	cis-Verbenol	11.961	0.13
10	Limonene diepoxide	13.042	0.09
11	α-Terpineol	14.058	0.18
12	Verbenone	14.538	0.13
13	Trans-carveol	15.111	0.04
14	Linalyl formate	16.142	0.04
15	Bornyl acetate	17.595	0.11
16	Germacrene B	19.424	0.14
17	β-Cubebene	21.783	0.04
18	β-Maaliene	22.362	0.30
19	Longifolene	23.166	0.06
20	γ-Muurolene	25.403	0.22
21	α-Gurjunene	27.816	0.04
22	β-Caryophyllene oxide	29.852	0.16
23	Humulene oxide II	30.676	0.23
24	Guaiene	31.418	0.19
25	Cadinol	32.115	0.18
	TOTAL		3.36

Also, several peaks remained unidentified, due to the absence of satisfying correspondence in the database (5.668, 16.926, 27.732, 27.422, 28.733, 29.037, 31.063, 32.630, 36.643, 38.752, 39.850, 45.664, 49.208, 50.663, 51.242, 52.028 and, respectively at 52.257 min.) representing the difference up to 100% (13.59%).

**Fig. 3:**
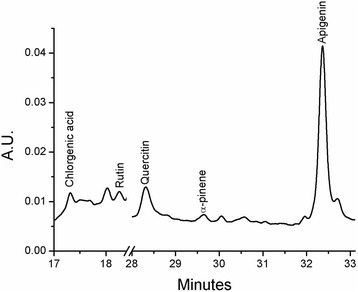
HPLC chromatogram of the juniper extract, presenting the selected compounds

The HPLC (chromatogram presented in Fig. 3) analyses were performed in order to further characterise the extract, considering three types of compounds: terpenoid (α-pinene), phenolic acid (chlorogenic acid) and flavonoids (rutin, apigenin, quercitin). The results revealed the presence of the selected compounds in the following concentrations: α-pinene – 22.60 ± 0.32 mg L^−1^, chlorogenic acid – 6.8 ± 0.15 mg L^−1^, rutin – 67.4 ± 0.81 mg L^−1^, apigenin – 13.2 ± 0.24 mg L^−1^, quercitin – 11.2 ± 0.22 mg L^−1^.

### Evaluation of the extract’s properties

#### The evaluation of antioxidant activity

The antioxidant activity of the extract was evaluated following the DPPH radical scavenging assay. The antioxidant potential of the crude extract was found to be 81.63 ± 0.38% (measured by the DPPH method, acc. Eq. (2)). The calculated EC_50_ (half maximal effective concentration) (1.42 ± 0.11 mg/mL) reveals a good antioxidant activity of the tested extract. The results were compared with the ones obtained for a known antioxidant (ascorbic acid), obtaining an EC_50_ value of 0.365 ± 0.006 mg/mL.

#### The evaluation of the antifungal activity

The diameters of inhibition zones (in millimetres) against test strains (*Aspergillus niger* ATCC 15475 and *Penicillium hirsutum* ATCC 52323) are presented in Table 5, for the tested extract, positive and negative controls, revealing a good antifungal activity of the juniper extract.

**Table 5:** The results of the antifungal activity assay (inhibition zones, in millimetres), including statistical interpretation^*^

Fungal line	Negative control (mm)	Extract (mm)	Positive control (mm)
*Aspergillus niger*	8.03 ± 0.0839^c^	20.9 ± 0.11^b^	41.6 ± 0.35^a^
*Penicillium hirsutum*	8.1 ± 0.0333^c^	17.2 ± 0.0882^b^	39.4 ± 0.42^a^

^*^Values represent *means ± SEM*; values in a row without a common superscript letter are statistically different (*P* < 0.05) as analysed by one-way ANOVA and the Tukey test

#### The evaluation of genoprotective properties

**Fig. 4:**
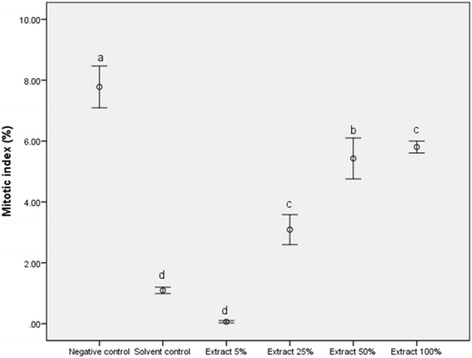
Mitotic indices induced by hydro-alcoholic extracts of *J. communis* L. in root tip cells of *A. cepa* L. (bars represent the standard error; a, b, c, d: the interpretation of the significance of the differences by means of the Duncan test, p˂0.05)

The effects of juniper hydro-alcoholic extracts on cell division in the root tips of *Allium cepa* L are presented in Fig. 4. Significant lower MI values than that of the negative control were induced by all tested concentrations of hydroalcoholic extracts. In our study the lowest MI values were induced by the extract’s solvent, water-ethanol 1:1 respectively. A strong mitotic delay induced by low concentrations of ethyl-alcohol in a short treatment time was noticed by Arcara and Nuti-Ronchi [42]. In this context, it is important to notice that comparing with the solvent used as positive control, a significant higher MI values were noticed for hydroalcoholic extracts at concentrations of 25%, 50% and 100%, suggesting the mitostimulatory effects of *J. communis* L. extracts.

**Fig. 5:**
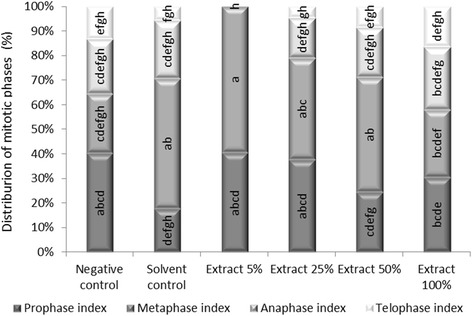
The influence of the juniper extract on the distribution of the mitotic phases in the root cells of *Allium cepa* L. (a–h: interpretation of the significance of the differences, by means of the Duncan test, *p* < 0.05)

Changes in the mitotic phase index (Fig. 5) were observed together with changes in MI after 48 h of incubation in each concentration tested.

**Fig. 6:**
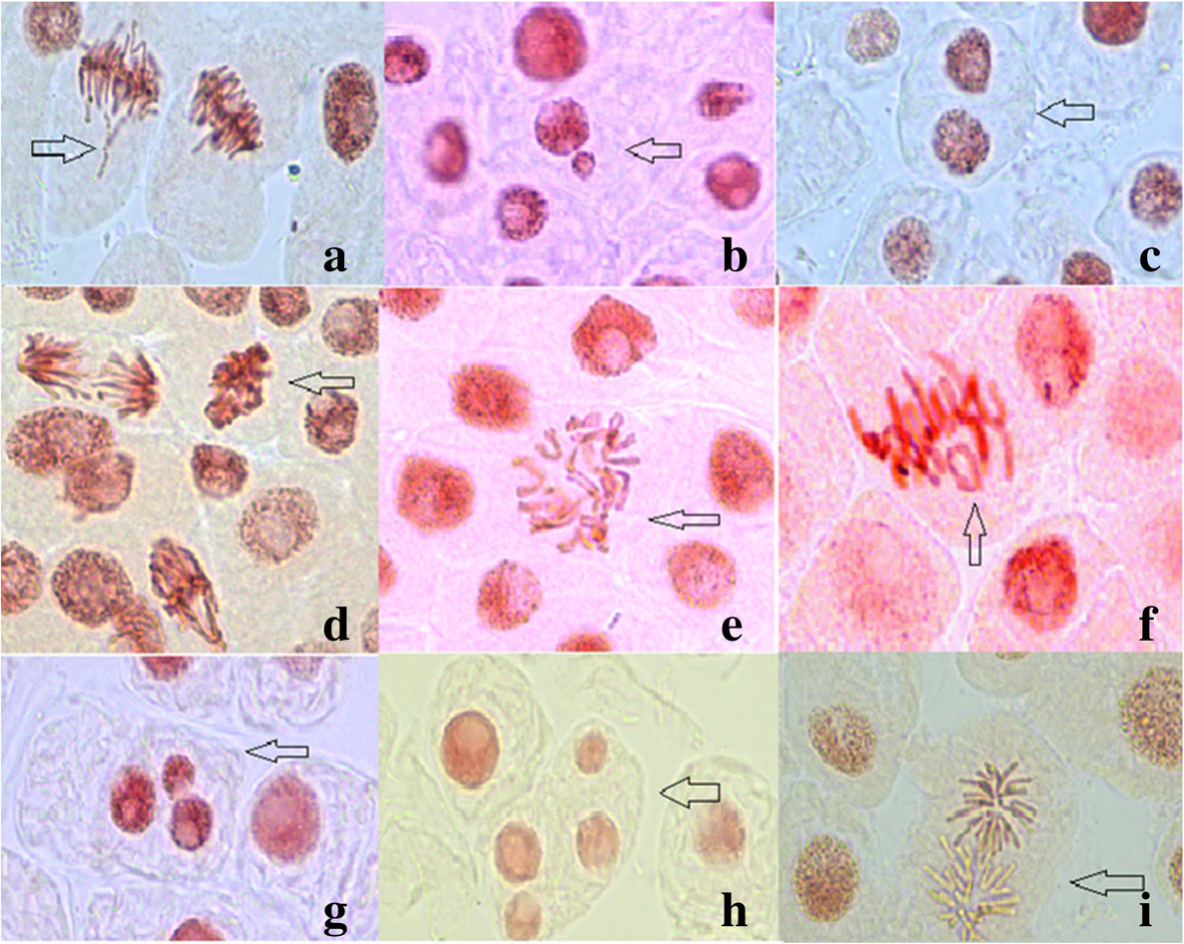
Chromosome aberrations and mitotic abnormalities induced by the solvent and 5% hydro-alcoholic extract of *Juniperus communis* L. **a** – laggards, **b** – micronucleus, **c** – binucleated cell, **d** – stickiness, **e** – C-mitosis, **f** – ring chromosome, **g**, **h** – multinucleated cells, **i** – star anaphase

Table 6 presents the effects of different concentrations of hydro-alcoholic extracts of *J. communis* L. on the chromosomes/mitosis in *A. cepa* L. root tip cells. Chromosome aberrations such as laggards and sticky chromosomes, and mitotic abnormalities such as micronuclei, binuclear cells and C-mitosis (Fig. 6) were observed in a higher frequency in the meristematic root cells incubated in the solvent control and hydro-alcoholic extract 5%.

**Table 6:** Types and frequencies of chromosome aberrations and mitotic abnormalities induced by the hydro-alcoholic extracts of *Juniperus communis* L^*^

Treatment	Chromosome/mitotic abnormalities (%)					
	Laggards	Micronuclei	Binucleated cells	Stickiness	C-mitosis	Other abnormalities
Negative control	13.43 ± 3.77^b^	–	–	–	–	–
Solvent control	10.87 ± 1.77^b^	0.24 ± 0.02^b^	–	–	12.38 ± 9.04^b^	3.55 ± 2.22^b^
5% extract	–	0.83 ± 0.37^b^	0.73 ± 0.29^b^	–	25.56 ± 10.24^a^	–
25% extract	–	0.11 ± 0.02^b^	0.35 ± 0.23^b^	10.56 ± 4.77^b^	29.31 ± 15.34^a^	–
50% extract	–	0.03 ± 0.03^b^	0.37 ± 0.12^b^	–	12.63 ± 11.69^b^	–
100% extract	–	–	0.22 ± 0.11^b^	–	4.34 ± 2.20^b^	–

^*^a, b: the interpretation of the significance of the differences by means of the Duncan test, *p*˂0.05

#### The evaluation of anti-inflammatory properties

In vitro pharmacological effects of polyphenolic compounds are numerous [36, 43], but the in vivo success appears to be limited by their poor bioavailability [37]. Therefore, the juniper extract was formulated as a microemulsion and tested for its anti-inflammatory action.

In order to evaluate the optimum microemulsion composition, different component ratios from the microemulsion were selected for further characterization. The microemulsion with the highest stability and lowest mean droplet size was selected and used in the experiments. The selected formulation contained 5% *Juniperus communis* L. extract, 9.29% oleic acid, 57.14% Tween 80: PG 1:1 (*w*/w) mixture and 28.57% water.

The droplet size was 134 ± 7 nm, and was not significantly affected by incorporation of the extract when compared to the droplet size of microemulsion alone. Also, no significant diameter change was found after 3 months of storage at 25 °C.

Zeta potential was observed to be −39.3 ± 3.7 mV, also a good indicator of a stable formulation. The high conductivity (163 ± 3.4 μS/cm) revealed the o/w structure of the microemulsion. The refractive index varied between 1.32 and 1.37 over 3 months, showing that the prepared microemulsion remained transparent and clear even after long-term storage. The pH ranged between 4.9 and 5.1 in this time interval.

The anti-inflammatory effect in the two inflammation experimental models used (acute-dextran and subacute-kaolin) [44] was studied by the plethysmometry method, using two control groups (untreated rats and rats treated with the microemulsion vehicle) and a reference substance (diclofenac). The results of the plethysmometric measurements were statistically analysed in order to obtain paw oedema evolution for the groups with dextran- and kaolin-induced inflammation, respectively. These data are summarized in Tables 7 and 8.

**Table 7:** The results of the plethysmometric measurement of the paw oedema induced with dextran, including statistical interpretation (V, the volume of oedema, mL; t Test, p)

Experimental group	V0	V1 h	V2 h	V3 h	V4 h	V5 h	V24 h
Negative control	1.19 ± 0.07	2.02 ± 0.15	2.01 ± 0.14	2.15 ± 0.17	2.11 ± 0.15	2.07 ± 0.10	1.43 ± 0.10
	*p*	<0.05	<0.05	<0.05	<0.05	<0.05	<0.05
Positive control	1.11 ± 0.08	1.76 ± 0.23	1.91 ± 0.17	1.83 ± 0.14	1.85 ± 0.19	1.89 ± 0.15	1.32 ± 0.11
	*p*	<0.05	<0.05	<0.05	<0.05	<0.05	<0.05
Treated with diclofenac	1.13 ± 0.09	1.71 ± 0.18	1.70 ± 0.16	1.79 ± 0.14	1.86 ± 0.15	1.72 ± 0.19	1.40 ± 0.22
	*p*	<0.05	<0.05	<0.05	<0.05	<0.05	<0.05
Treated with microemulsion	1.13 ± 0.11	1.72 ± 0.13	1.77 ± 0.13	1.82 ± 0.13	1.83 ± 0.09	1.75 ± 0.20	1.31 ± 0.11
	*p*	<0.05	<0.05	<0.05	<0.05	<0.05	<0.05

**Table 8:** The results of the plethysmometric measurement of the paw oedema induced with kaolin, including statistical interpretation (V, the volume of oedema, mL; t Test, p)

Experimental group	V0	V1 h	V2 h	V3 h	V4 h	V5 h	V24 h
Negative control	1.18 ± 0.08	1.86 ± 0.10	1.89 ± 0.12	1.95 ± 0.11	2.01 ± 0.11	2.02 ± 0.07	1.96 ± 0.12
	*p*	<0.05	<0.05	<0.05	<0.05	<0.05	<0.05
Positive control	1.03 ± 0.08	1.36 ± 0.08	1.53 ± 0.14	1.54 ± 0.14	1.58 ± 0.15	1.42 ± 0.12	1.41 ± 0.12
	*p*	<0.05	<0.05	<0.05	<0.05	<0.05	<0.05
Treated with diclofenac	1.14 ± 0.07	1.71 ± 0.17	1.82 ± 0.18	1.83 ± 0.13	1.90 ± 0.24	1.86 ± 0.11	1.96 ± 0.18
	*p*	<0.05	<0.05	<0.05	<0.05	<0.05	<0.05
Treated with microemulsion	1.10 ± 0.09	1.42 ± 0.13	1.50 ± 0.16	1.52 ± 0.15	1.54 ± 0.18	1.58 ± 0.23	1.92 ± 0.17
	*p*	<0.05	<0.05	<0.05	<0.05	<0.05	<0.05

The juniper-containing microemulsion presented a similar paw oedema evolution to the group treated with diclofenac at all the measurement times (t test, *p* > 0.05).

## Discussion

Natural products obtained from the plant kingdom (either as extracts or essential oils) represents very complex mixtures, having applicability in various areas, such as food, beverages, flavouring and aroma agents or in several medical applications (as anticancer, anti-diabetic, antibacterial, antiviral and antioxidant agents), mainly due to their polyphenolic compounds content [1–8].

Potential medical applications of *Juniperus communis* L. are related to its ethnomedicinal use as diuretic, in gastrointestinal diseases, renal, genital, pulmonary and rheumatic disorders [11]. Recent scientific papers describe the potential use of juniper natural products as antioxidant [12], antimicrobial [13, 14] or hypoglycaemic and hypolipidemic agents [15].

The anti-inflammatory potential of different types of juniper extracts (alcoholic, aqueous or hydro-alcoholic) are usually documented in large survey studies, evaluating different medicinal plants specific to each author’s native region [16–19]. Depending on the solvent used for extraction and specific characteristics of the plant material used, different authors found juniper extract to be active, with an anti-inflammatory potential ranging from average to very good.

The present study was designed considering two main aspects: characterisation of the hydro-alcoholic extract and evaluation of its potential applications. The evaluation of the extraction efficiency and the phytochemical assays offers a first glance on the extract composition. The polyphenolics content it’s comparable with literature data regarding European native juniper extracts (Kurti et al. [24] reported values ranging from 4.7 to 5.83 mg g^−1^ dry plant weight for ethanol extracts obtained from 20 localities in the Republic of Macedonia, while Miceli et al. [45] reported values of 17 to about 60 mg GAE g^−1^ extract for methanol extracts of *Juniperus communis* L. var. *communis* and *Juniperus communis* L. var. *saxatilis* Pall) as is also the case for the total flavonoids content. The results of the phytochemical assays suggest a high mono-terpenoid content, that could be an indicator of a very good antioxidant potential [30]. Due to the fact that the terpenoid content varies strongly with the time of harvesting, growing area and other factors [44], it is difficult to compare samples of different origins. The GC-MS and HPLC analyses completes the evaluation of the extract’s composition.

The results presented herein support the application of juniper hydro-alcoholic extract as antioxidant, antifungal and anti-inflammatory agent. The calculated EC_50_, comparable with literature data presented, for example, by Miceli et al. [45] (values ranging from 0.63 to 1.84 mg mL^−1^) are in a good concordance with the high mono-terpenoid content established by the phytochemical assay.

The antifungal potential of the extract (that can be correlated with its phytochemicals content) is very good, compared with literature data [46–49]. However, a superior effect is observed on *A. niger,* if compared with the positive control (for *A. niger,* the inhibition zone for the sample represents 41.46% of the inhibition zone of the positive control, while for *P. hirsutum*, only 20.5%).

The cytogenetic study reveals an increase in the percentage of metaphase cells was noticed for the experimental variants characterized by the incubation of roots in solvent and hydro-alcoholic extract 5%. This mitodepressive process was clearly evident for the other dilutions, but was significant inhibited by the undiluted hydro-alcoholic extract. These indices reflect stimulation of metabolic activity in meristematic root cells of *A. cepa* L. incubated with juniper hydroalcoholic extracts. Moreover, when meristematic root cells were incubated in solvent control for 48 h, microscopic slides analysis inconsistently revealed other abnormalities such as ring chromosomes, polar star anaphase and multinucleated cells (as presented in Fig. 5). The sensitivity of plant cells to the clastogenicity of ethanol (micronuclei induction, chromosome damage and SCEs) were extensive presented in the review of Phillips and Jenkinson [50].

Although frequency of the observed abnormalities was not significantly different from the negative and positive controls, it can be noticed a serious decrease of the abnormalities types and frequencies for the 100% extract. The results demonstrate the genoprotective potential of *J. communis* L. undiluted extract, inhibiting the mitodepressive effect of ethanol. Capacity of juniper extract to mediate chromosome damage induced by ethanol in root tip cells of *A. cepa* L., may be attributed to its high antioxidant activity revealed by the DPPH radical scavenging assay.

As previously mentioned, the study of the pharmacological effect of polyphenolics in vivo is severely limited by their poor bioavailability, as presented by Mahmood et al. [37]. Thus, the study was performed using juniper extract formulated as microemulsion. Considering the in vivo determination of the anti-inflammatory effect, for the dextran-induced inflammation model, the paw oedema volume was significantly higher compared to the initial values (*p < 0.05*) for all animal groups, without return to baseline at the end of the observation period (as presented in Table 7). The global paw oedema evolution process for the groups treated with microemulsion was different from the negative control group and the group treated with diclofenac (ANOVA, *p < 0.05*). Dextran generates an osmotic oedema caused by the mast cells degranulation with the release of histamine and serotonin and increase of vascular permeability [51, 52]. The decrease of the pow oedema of the animals treated with the juniper microemulsion indicates anti-histamine and anti-serotine activity properties, in the dextran experimental model used. Studies indicates that chlorogenic acid [53] and quercitin shows inhibitory effect on mast cells degranulation [54].

The microemulsion vehicle influenced the global paw oedema evolution process (ANOVA, *p < 0.05*), but the process was similar to the negative control group at the measurement times (t test, *p > 0.05*).

For the kaolin-induced inflammation model, the paw oedema volume was significantly higher compared to the initial values (*p < 0.05*) for all the animal groups, without return to baseline at the end of the observation period (Table 8). In the kaolin model, the inflammatory response it appears to be increased, at the end of the experiment, for the animals treated with juniper extract microemulsion that might be explained by a short half-life of the extract that might be correlated with the exerted a maximum anti-inflammatory effect in the first 4 h of the experiment. Those data suggest that for obtaining a prolonged anti-inflammatory effect it is necessary a multiple dose administration regimen.

For the kaolin-induced inflammation model, the global paw oedema evolution process for the groups treated with juniper-containing microemulsion was different from the negative control group and the group treated with diclofenac (ANOVA*, p < 0.05*). The microemulsion vehicle did not influence the global paw oedema evolution process, the process being similar to the negative control group (ANOVA, *p > 0.05*). The microemulsion had a similar paw oedema evolution after 24 h with the group treated with diclofenac (t test, *p > 0.05*). The animals treated with the juniper-containing microemulsion exerted a maximum anti-inflammatory effect in the first 4 h of the experiment.

The kaolin induced inflammation model lead to the increase of proinflammatory cytokines e.g. f IL-1β, IL-6 and TNFα [55]. Studies showed that flavonoids [56, 57] and quercitin [58, 59] downregulates the expression of proinflammatory cytokines. The obtained results indicates activity of the juniper microemulsion against proinflammatory cytokines, in the kaolin experimental model used.

The results presented herein establishes the anti-inflammatory action of the juniper extract administered as microemulsion in both models, with increased activity when compared to kaolin subacute inflammation induced model. However, the optimal dose levels and the mechanism of action are uncertain, and the active chemical compounds responsible for the anti-inflammatory activity of the juniper extract needs further experiments in order to be completely clarified and to be able to translate the obtained results to other species or systems. The literature data proposes a synergistic action of multiple compounds, and does not attribute the biological effects (antioxidant, antifungal, or anti-inflammatory) to a single compound [60–64]. Many authors propose correlations between phenol/flavonoid content and the anti-inflammatory action [65, 66]. Our results indicate the possibility of developing the extract into a potent, lower-cost and safer therapeutic agent, compared with currently used synthesised agents.

## Conclusions

The data obtained in the present this study demonstrates the genoprotective, antioxidant, antifungal and anti-inflammatory properties of the hydroalcoholic extract obtained from juniper berries native to Romanian southern sub-Carpathian hills. The extract shows mitogenic and genoprotective effects, that could also indicate its immunostimulatory effects and its potential as cellular metabolic regulator. The anti-inflammatory effect obtained after the administration of juniper extract as microemulsion may recommend this formulation for further studies as dietary factors in pathologies with inflammatory component.
